# Radical Nephrectomy Following Prior Cryoablation for Renal Cell Carcinoma: A Case Report and Literature Review

**DOI:** 10.1002/iju5.70127

**Published:** 2025-12-31

**Authors:** Spyridon Mitsios, Panagiotis Balaxis, Athanasios Klampatsas, Anastasia Nikolaidou, Gkatzos Stergios, Panagiotis Kousidis, Evangelos Petsatodis, George Moustakas

**Affiliations:** ^1^ Department of Urology Theageneio Cancer Hospital of Thessaloniki Thessaloniki Greece; ^2^ Department of Diagnostic Pathology Theageneio Cancer Hospital of Thessaloniki Thessaloniki Greece; ^3^ Interventional Radiology Department G. Papanikolaou General Hospital Thessaloniki Thessaloniki Greece

**Keywords:** ablation techniques, carcinoma, cryosurgery, nephrectomy, recurrence, renal cell

## Abstract

**Introduction:**

Small renal masses account for 48%–66% of renal cell carcinoma diagnoses, influencing management decisions for low‐stage kidney disease. This report presents a case of cancer recurrence and tumor progression into the renal pelvis postcryoablation, managed with radical nephrectomy.

**Case Presentation:**

A 70‐year‐old woman with a history of cryoablation for T1_b_ renal cell carcinoma in the right kidney presented with persistent hematuria. Imaging revealed local recurrence and carcinoma invasion of the renal pelvis. The patient underwent open radical nephrectomy. Intraoperatively, the kidney was found adhered to the peritoneum and vena cava. The remaining elements of renal, hilar and adrenal anatomy showed no abnormalities. The postoperative course was uneventful.

**Conclusion:**

While local recurrences following cryoablation can often be safely retreated with cryoablation, progression involving the renal pelvis is rare and demands heightened vigilance and expertise. Tailored approaches, including radical nephrectomy and technical adaptability, are essential for achieving optimal oncological and functional outcomes.

**Abbreviations**
CT: computed tomography
EUA: European urology association
GFR: glomerular filtration rate
ISUP: international society of urological pathology
MRI: magnetic resonance imaging
NCCN: National comprehensive cancer network
PUJ: pelvic ureteric junction
RCC: renal cell carcinoma
SRMs: small renal masses
US: ultrasound
WHO: World health organization

**Keynote Message**

Renal cell carcinoma recurrence after cryoablation is rare but typically occurs within 2 years. Management varies, with repeat ablation or partial nephrectomy for small tumors and radical surgery for more extensive disease. Herein we report a case of recurrence with renal pelvic invasion that required radical nephrectomy.

## 1 Introduction

RCC is increasingly detected at earlier stages due to advanced imaging techniques, resulting in a rise in diagnoses of small renal masses (≤ 4 cm), now accounting for 48%–66% of new RCC diagnoses [1]. The standard approach for SRMs has traditionally been partial nephrectomy, aiming to preserve renal function and reduce morbidity [2, 3]. Cryoablation has emerged as a less invasive alternative for select renal masses (T1b ≤ 7 cm in greatest dimension), offering comparable oncologic outcomes in carefully selected patients [4, 5, 6, 7].

## 2 Case Presentation

**FIGURE 1:**
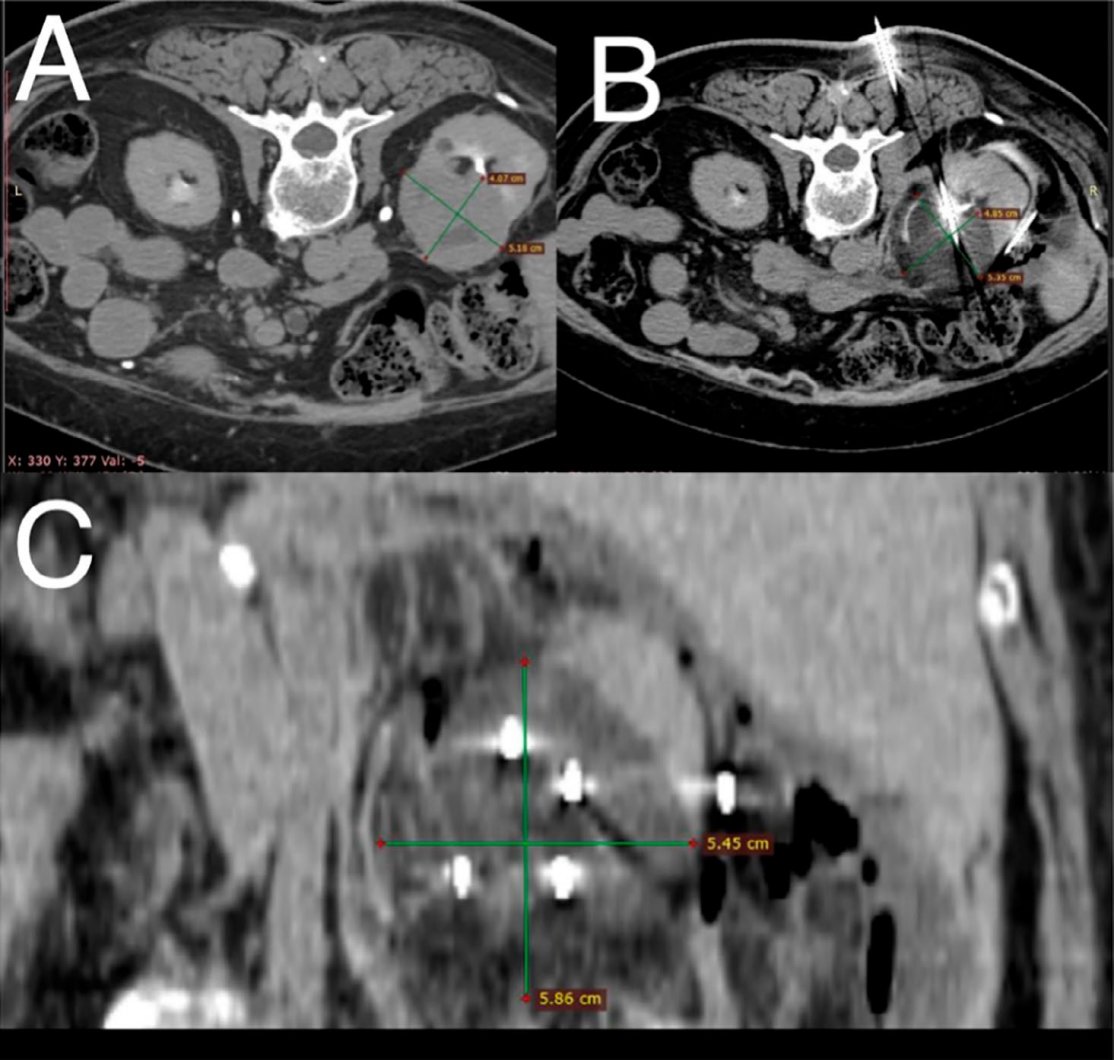
CT images before (A) and during (B) the cryoablation procedure. The hypodense iceball (C) created by multiple cryoprobes is completely covering the tumor, creating adequate margins. The intestine and the ureter were hydrodissected in order to perform a safe ablation.

**FIGURE 2:**
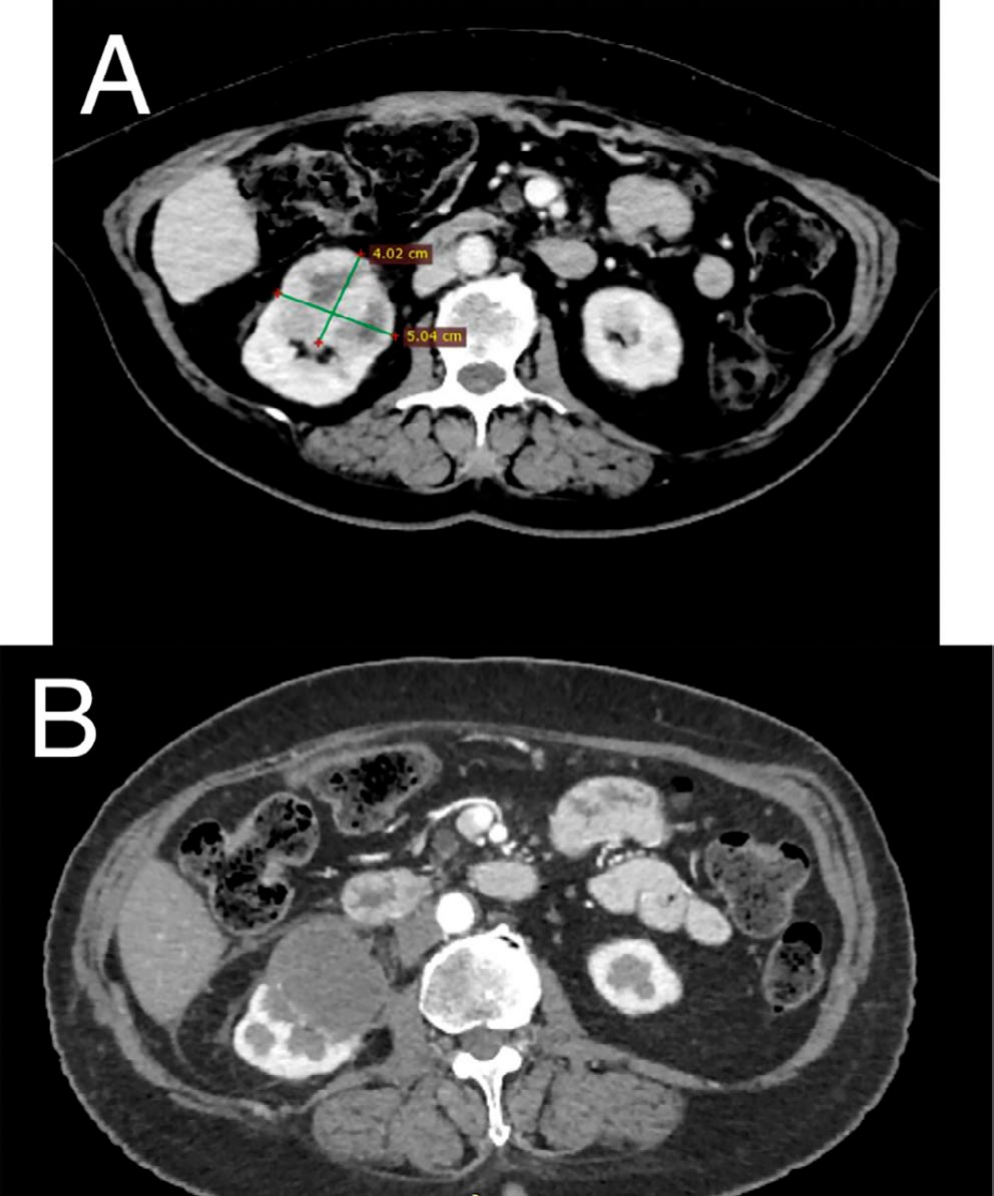
CT images before (A) and 6 months after (B) cryoablation procedure show complete tumor necrosis with no visible enhancement after contrast medium administration.

A 70‐year‐old female patient presented with persistent gross hematuria. The patient had a history of prior cryoablation, for T1b clear cell RCC in the right kidney 2 years earlier, arterial hypertension and paraneoplastic thrombocytosis. Initial precryoablation CT revealed a 5 cm tumor (T1b) without renal pelvis invasion. Based on a R.E.N.A.L. nephrometry score of 10 ph, partial nephrectomy complexity was high with a 21.9% likelihood of major complications [8]. Following multidisciplinary oncology tumor board review, cryoablation was selected as a less invasive procedure. Intraoperative CT showed that the “iceball”—the visible ablation zone of frozen tissue forming around the cryoprobes—contained the entire tumor (Figure 1). No major complications occurred during or after cryoablation. Six‐month follow‐up CT showed complete necrosis and devascularization (Figure 2).

**FIGURE 3:**
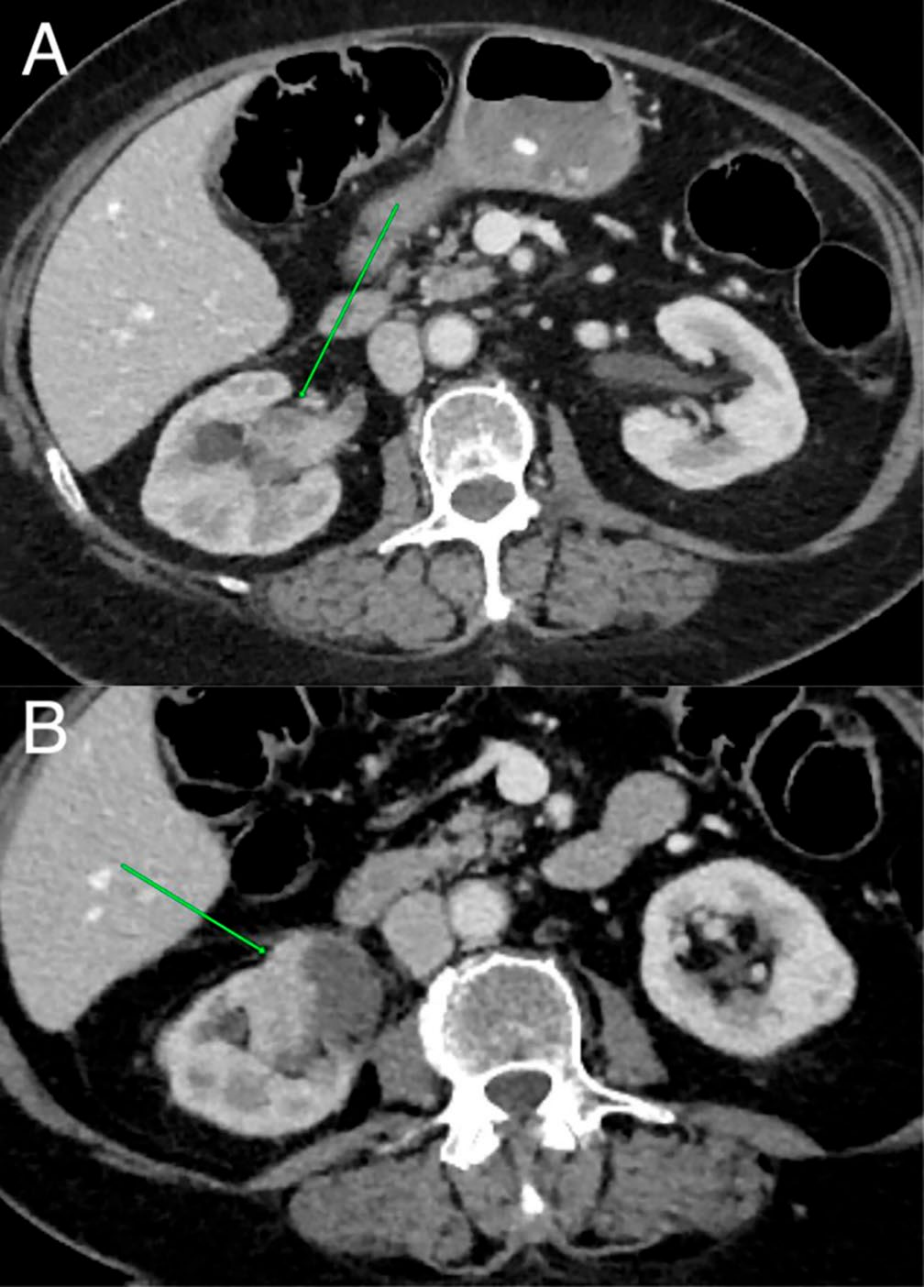
Abdominal computed tomography 2 years after cryoablation. A viable tumor is depicted expanding into the renal pelvis (A) as well as the periphery of the previously ablated tumor (B).

The current CT scan 2 years postablation demonstrated recurrence at the periphery of the previous ablation zone near the renal hilum and invasion of the right renal pelvis (Figure 3). ^99m^Tc‐DTPA Renal Dynamic Imaging showed minimal GFR reduction in both kidneys, unchanged from precryoablation results with near‐equal contribution of both kidneys to total function. The patient was scheduled for open right radical nephrectomy.

**FIGURE 4:**
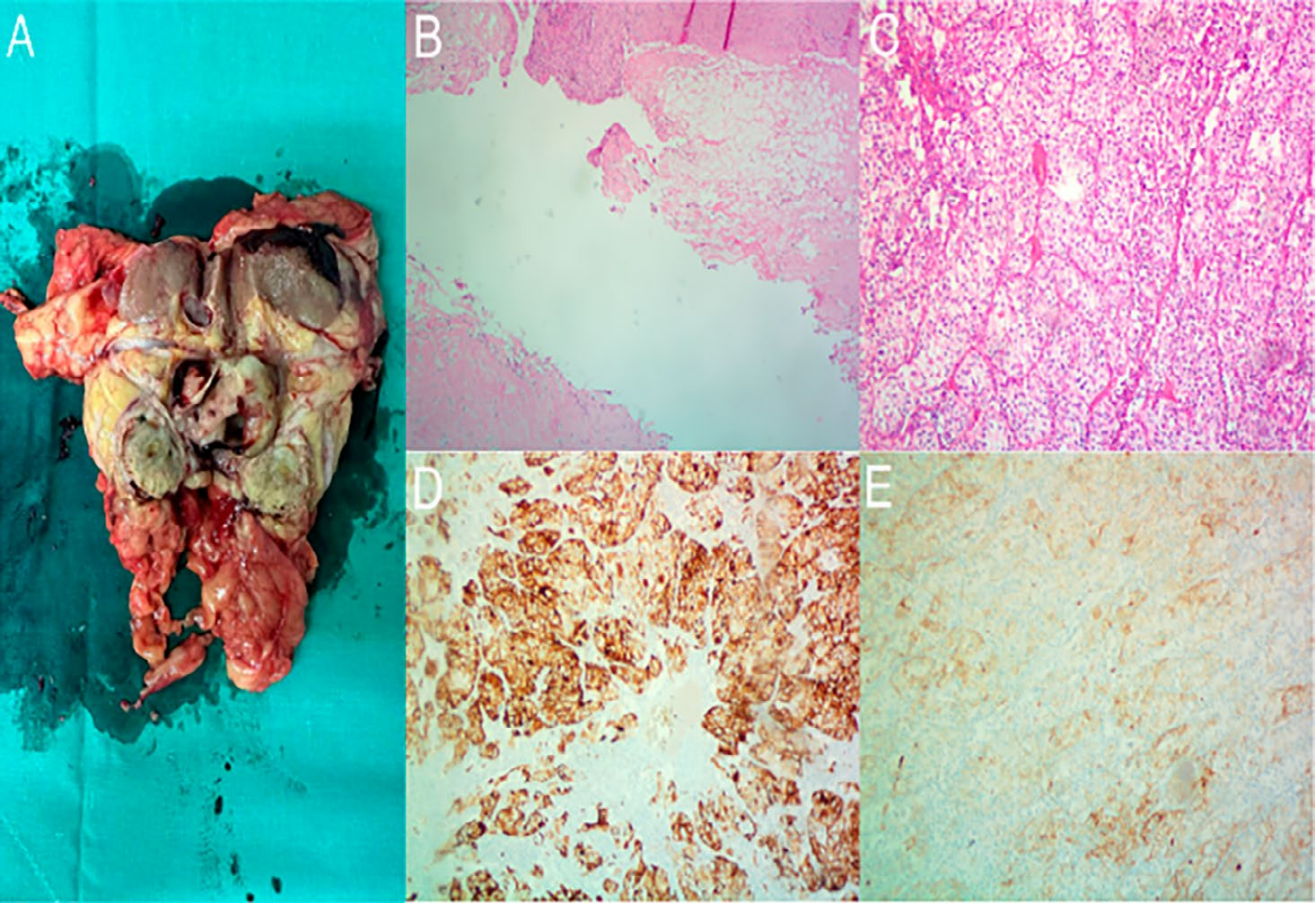
Postoperative pathological findings (A) Macroscopic features of the tumor: In the lower pole of the kidney, a whitish polypoid tumor is identified, measuring 5 × 3.3 × 2 cm, which appears to project into the renal pelvis (B) Hematoxylin–eosin staining showing clear cell renal cell carcinoma, 10× magnification. (C) Invasion of the carcinoma into renal pelvis, hematoxylin–eosin staining, 4× magnification. (D) Immunohistochemical staining positive for Keratin AE1/AE3, 10× magnification. (E) Immunohistochemical staining positive for CD10, 10× magnification.

Intraoperatively, the kidney was adherent to the peritoneum, vena cava and adjacent structures. Adhesiolysis was performed. During kidney mobilization, hemorrhage from a vein draining into the vena cava was identified and successfully ligated with sutures. The remaining anatomical elements of the kidney, adrenal gland and vessels showed no anatomical differentiations. Surgery proceeded with careful mobilization, hilar ligation, and en bloc resection of the kidney, adrenal gland and Gerota's fascia. Post operative course was uneventful. Pathology (Figure 4) revealed a 5 × 3,3 × 2cm clear cell RCC with variable nuclear grade of malignancy (predominantly grade 1 and 2, with focal grade 3 areas) according to WHO/ISUP. The tumor invaded the renal capsule without perinephric fat infiltration and had emerging infiltration of the renal pelvis.

## 3 Discussion

According to the latest EUA guidelines (March 2025 update) for localized RCC multiple treatment methods exist. For T1 tumors partial nephrectomy should be offered if technically feasible (strong evidence level). Radical nephrectomy is appropriate when partial nephrectomy is not feasible by any approach. Regarding cryoablation, it can be offered to frail and/or comorbid patients with small renal masses (T1) per EUA guidelines [9]. Conversely, NCCN guidelines permit ablative therapies for T1 tumors [10]. For T1a tumors, partial nephrectomy is preferred and percutaneous ablation is acceptable, while for T1b tumors, partial nephrectomy is the treatment of choice if technically feasible with ablative methods offered only in carefully selected patients when surgery is contraindicated [10]. In this case, following multidisciplinary oncology tumor board review, the serious comorbidities of the patient and the high R.E.N.A.L. nephrometry score made surgical resection high‐risk and clinically unsuitable, necessitating an ablative approach. Following cryoablation, the patient underwent follow‐up with bloodwork including creatinine and blood urea nitrogen, plus chest CT and triphasic renal CT at 1, 3, 6, and 12 months, then yearly thereafter. NCCN recommends that postablation imaging follow‐up consist of abdominal CT or MRI with and without intravenous contrast, or contrast‐enhanced US at 1–3 months, 6 months, and 12 months after ablation, then annually thereafter as well as chest imaging with x‐ray or CT annually for 5 years for patients with biopsy‐proven low‐risk pathologic features (no sarcomatoid, low‐grade [grade 1/2] RCC), nondiagnostic biopsies, or no prior biopsy [10]. Most local recurrences occur within 10–20 months [11]. Rates after 3 years range from 2%–3% [12]. Conversely, other studies demonstrate that up to 12.5% of the patients previously treated with focal therapy require repeat or salvage procedures [13]. A systematic review of 347 patients with T1b tumors reported local recurrence rates that ranged from 2.8% to 27%; however, primary and secondary success rates were high at 84%–98% and 92%–100%, respectively [7]. Further evidence suggests local recurrence is more common after focal ablations than surgical resections, although the majority of these recurrences can be managed with a second attempt at ablation [14, 15]. Multiple management options exist after local recurrence. Active surveillance may be a safe option for up to a year in cases with early enhancement after cryoablation, as this may reflect postoperative inflammation [14]. Repeated cryoablation is the most commonly performed procedure after failed ablation [14]. Studies report 0.9%–1.3% of cryoablated lesions undergo reablation [16, 17]. Salvage surgery is required for large tumors, or if repeat ablations fail [14]. Partial nephrectomy is ideal for renal function preservation but may be difficult due to fibrosis or scarring from prior ablation. Radical nephrectomy is needed in large or complex cases. Both open and minimally invasive salvage nephrectomies are described. Open surgery after cryoablation is technically demanding due to adhesions and remodeling with higher complication rates than in primary procedures [18]. Regarding cryoablation versus partial nephrectomy as first‐line treatments for small renal masses, a 2019 meta‐analysis by Deng et al. reported that cryoablation was associated with worse oncological outcomes, significantly reduced complication risk and improved kidney function preservation compared to partial nephrectomy [19]. For T1b tumors, Caputo et al. reported increased recurrence with cryoablation versus partial nephrectomy, though 1‐year survival outcomes were comparable [2]. In this case, recurrence may reflect inadequate iceball margin (4 mm vs. optimal 7 mm). Although Lipiodol marking was not used, multiple cryoprobes and hydro dissection were employed to ensure enhanced treatment potency and minimize energy loss during cryoablation (cold sink effect). Proximity to the renal pelvis may have acted as a heat sink, reducing the lethal efficacy of the iceball at tumor margins. Tumor biology may also have contributed. Notably, imaging during the first year showed complete remission.

## 4 Conclusion

Local recurrence postcryoablation is rare. For tumors 4–7 cm, cryoablation remains a viable option in select cases. Most recurrences occur within 2 years. No definitive guidelines exist for recurrence management. Cryoablation margins and long‐term outcomes in T1b tumors require further investigation. Repeat ablation or partial nephrectomy may treat small recurrences, while large recurrences necessitate radical nephrectomy.

## Consent

Informed written consent was obtained from the patient for publication of this case report and the accompanying images.

## Conflicts of Interest

The authors declare no conflicts of interest.

## Data Availability

The data that support the findings of this study are available from the corresponding author upon reasonable request.
